# Supplementary material for: Recipient tissue microenvironment determines developmental path of intestinal innate lymphoid progenitors
Source: Nat Commun. 2024 Sep 6;15:7809. doi: 10.1038/s41467-024-52155-2 (PMC11379955; doi:10.1038/s41467-024-52155-2)
Supplement: Supplementary file 1 — Supplementary Information [file 41467_2024_52155_MOESM1_ESM.pdf]

Supplementary Information

Recipient tissue microenvironment determines developmental path of intestinal innate lymphoid progenitors

Paula A. Clark[1*], Mayuri Gogoi[1†], Noe Rodriguez-Rodriguez[1†], Ana C. F. Ferreira[1], Jane E. Murphy[1], Jennifer A. Walker[1], Alastair Crisp[1], Helen E. Jolin[1], Jacqueline D. Shields[2] and Andrew N. J. McKenzie[1*]

[1] MRC Laboratory of Molecular Biology, Cambridge, CB2 0QH, United Kingdom.
[2] Translational Medical Sciences, School of Medicine, University of Nottingham Biodiscovery Institute, Nottingham, NG7 2RD, United Kingdom.

[†]Equally contributing authors

*Corresponding authors: Andrew N. J. McKenzie (anm@mrc-lmb.cam.ac.uk) and Paula A. Clark (pclark@mrc-lmb.cam.ac.uk)

This Supplementary Information File contains:

Supplementary Figures 1-12

Supplementary Table 1

Supplementary References (relating to Supplementary Figures)

Supplementary Figure 1

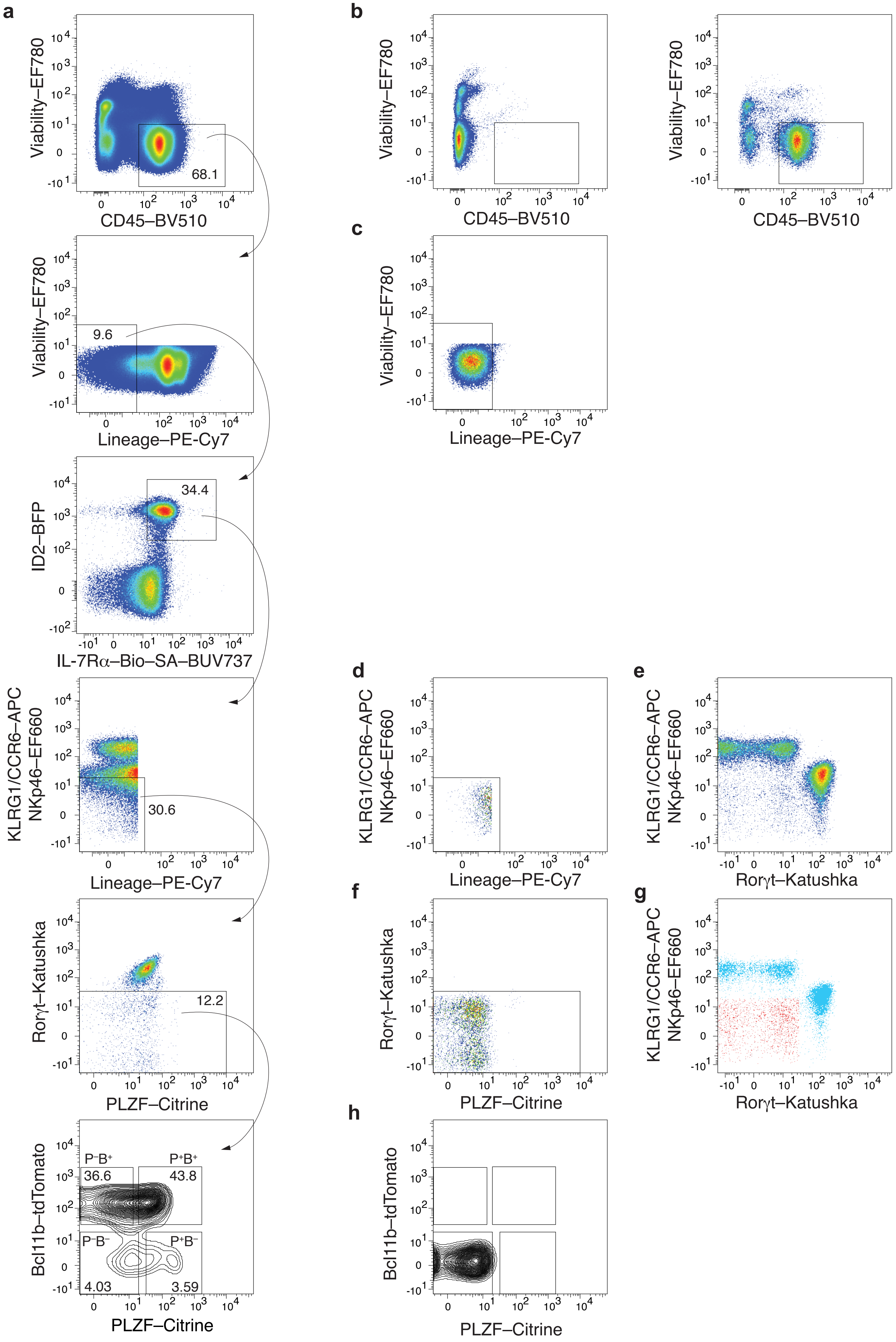

Supplementary Figure 1

FACS sorting strategy for mature marker negative siLP ILCs and siLP-ILCPs.

a) Representative flow cytometry plots of the sorting strategy for the $P^{+}B^{-}$, $P^{+}B^{+}$, $P^{-}B^{-}$ and $P^{-}B^{+}$ populations of siLP lymphocytes from four colour reporter mice, analysed in **Fig.1c-e**. Lineage = CD3, CD4, CD8, CD11b, CD11c, CD19, FcεR1, Ly6G/Ly6C, NK1.1, Ter119. Numbers indicate the percentage of the parent population each gate represents.

b) Representative flow cytometry plots of Viability-EF780 only, and Viability-EF780 and CD45-BV510 stained wildtype (WT) siLP cells to demonstrate definition of viable/CD45^{+} gate.

c) Representative flow cytometry plots of Viability-EF780 and CD45-BV510 stained WT siLP cells to demonstrate definition of lineage^{-} gate.

d) Representative flow cytometry plot of Viability-EF780, CD45-BV510 and lineage-PE-Cy7 stained WT siLP cells to demonstrate definition of KLRG1/CCR6/NKp46 negative gate.

e) siLP lymphocytes from four colour reporter mice stained with the full antibody panel plotted with KLRG1/CCR6/NKp46 against Rorγt-Katushka to demonstrate the double negative population.

f) WT viable, CD45 positive, lineage negative, IL7Rα positive, KLRG1/CCR6/Nkp46 negative siLP lymphocytes stained with the full antibody panel to demonstrate the definition of the Rorγt-Katushka negative gate.

g) Back gating of the cells defined in the sorting strategy as Rorγt-Katushka negative on to plot **(e)**.

h) WT viable, CD45 positive, lineage negative, IL7Rα positive, KLRG1/CCR6/Nkp46 negative siLP lymphocytes stained with the full antibody panel to demonstrate the definition of the Bcl11b-tdTomato and PLZF-Citrine gates.

This sorting strategy was used to purify siLP-ILCPs throughout the study.

Supplementary Figure 2

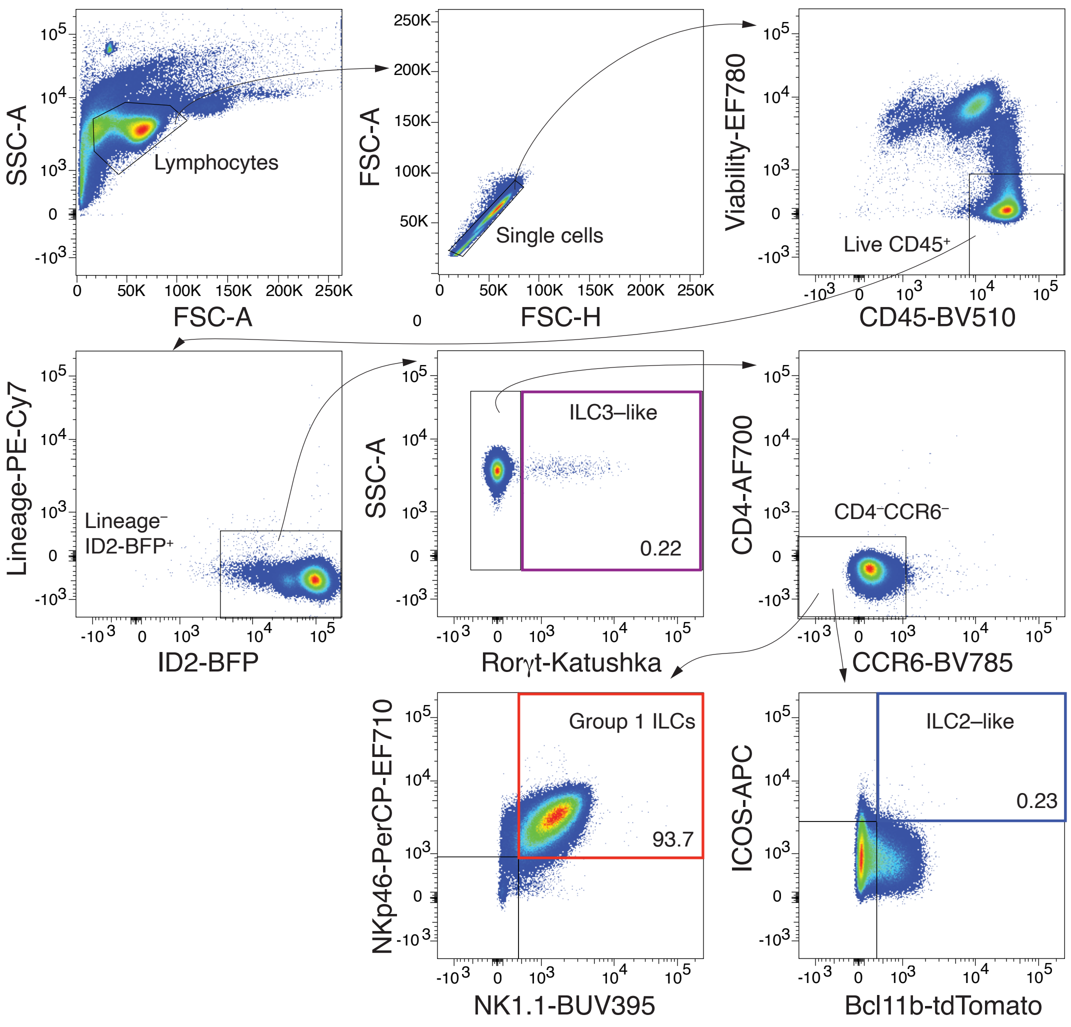

Supplementary Figure 2

Gating strategy for the phenotyping of P^+B^- in vitro progeny.

Representative flow cytometry plots for the gating strategy to define the P^+B^- in vitro progeny. Lineage = CD3, CD8, CD19, Ter119. Numbers within plots indicate the percentage of the parent population each gate represents. Group 1 ILCs cells are defined as $LiveCD45^+lineage^-Id2\text{-}BFP^+Ror\gamma t\text{-}Katushka^-CCR6^-CD4^-NKp46^+NK1.1^+$. The ILC2-like population is defined as $LiveCD45^+lineage^-Id2\text{-}BFP^+Ror\gamma t\text{-}Katushka^-CCR6^-CD4^-ICOS^+Bcl11b^+$. The ILC3-like population is defined as $LiveCD45^+lineage^-Id2\text{-}BFP^+Ror\gamma t\text{-}Katushka^+$. This gating strategy is used for the data in **Fig.1d and Supplementary Fig.3b**.

Supplementary Figure 3

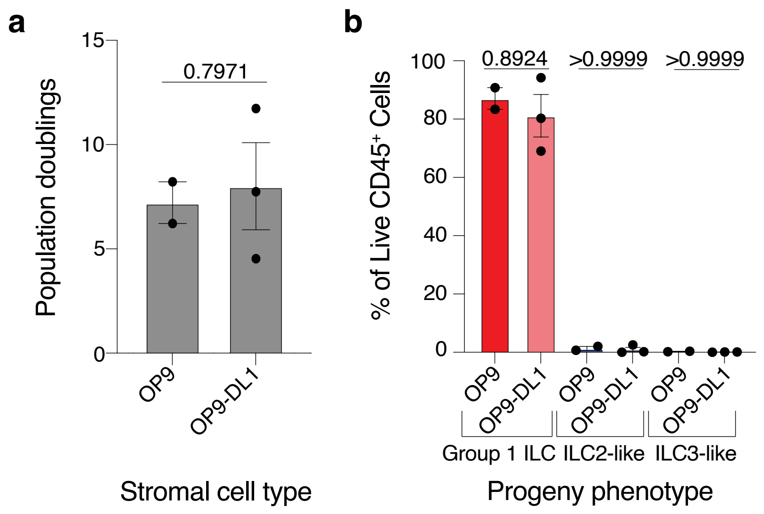

Supplementary Figure 3

Comparison of the numbers and phenotype of siLP-ILCP in vitro progeny on OP9 versus OP9-DL1 stromal cells

a) In vitro culture of the PLZF$^+$Bcl11b$^-$ (P$^+$B$^-$) siLP-ILCPs under neutral conditions (IL-7 and SCF) with either OP9 or OP9-DL1 stromal cells. Each data point represents the expansion of all the cells of an indicated phenotype from a single mouse. Since the numbers of cells sorted and seeded varies considerably between populations and mice this is expressed as population doublings. Data shown are from 1 experiment involving siLP-ILCPs from 2 mice with OP9 cells and 3 mice with OP9-DL1 cells (unpaired T test) and are representative of 2 similar independent experiments. Data plotted as mean with SEM error bars.

b) Proportions of ILC progeny produced from PLZF$^+$Bcl11b$^-$ (P$^+$B$^-$) siLP-ILCPs with either OP9 or OP9-DL1 stromal cells. **Supplementary Fig. 2** defines the phenotype of group 1 ILCs, ILC2-like and ILC3-like progeny. Data shown are from 1 experiment involving siLP-ILCPs from 2 mice with OP9 cells and 3 mice with OP9-DL1 cells. Data plotted as mean with SEM error bars and significance calculated by one-way ANOVA with Tukey's multiple comparisons test and are representative of 2 similar independent experiments.

Source data are provided as a Source Data file.

Supplementary Figure 4

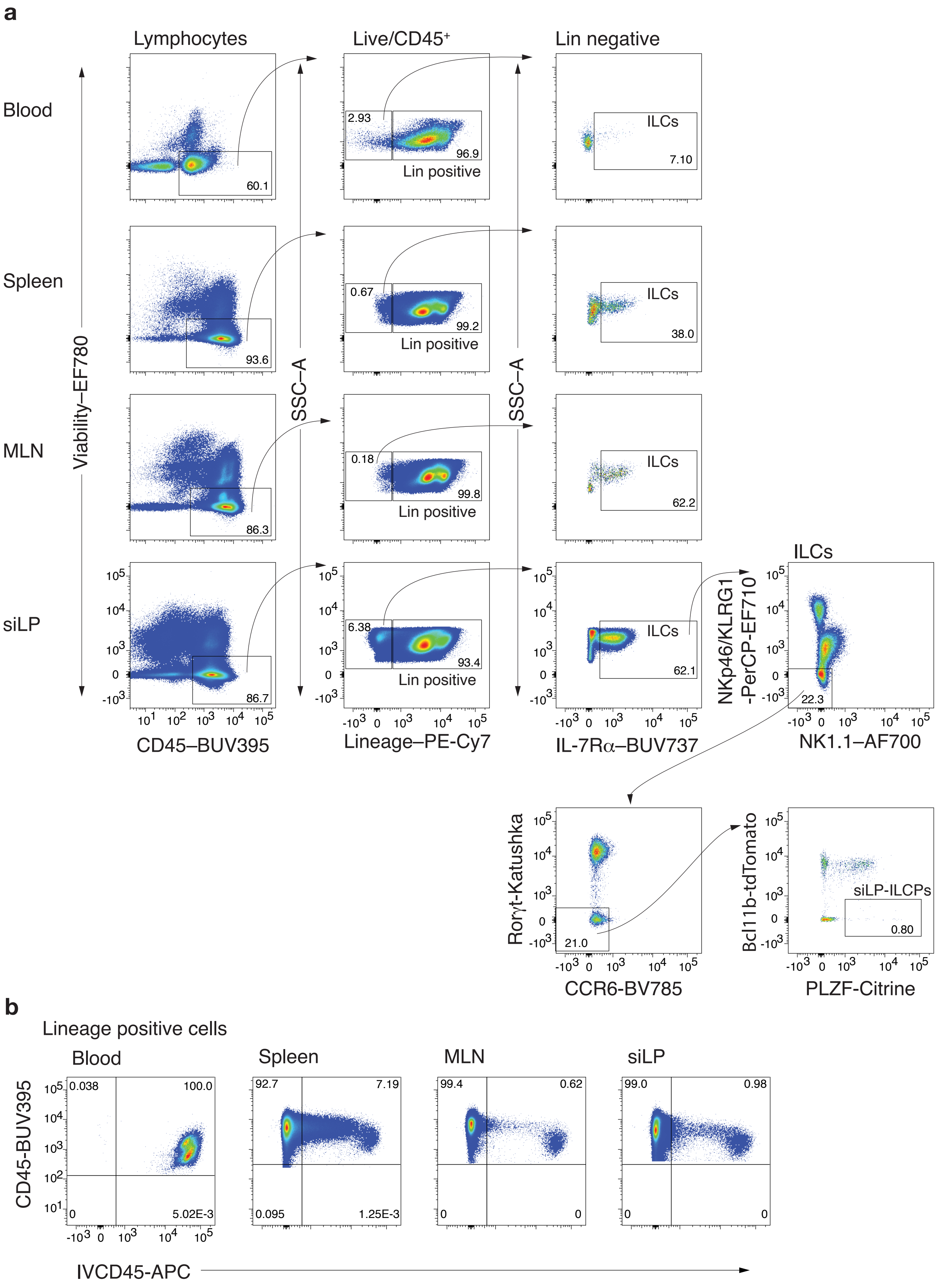

Supplementary Figure 4

Gating strategy for intravascular CD45 labelling analysis and ivCD45 labelling of lineage positive cells

a) Definition of lineage positive cells and ILCs across the indicated tissues, and ILCPs in the siLP only analysed in **Fig.2** and **Supplementary Fig.4b**. Numbers within gates indicate the percentage of the parent population each gate represents. Lineage = CD3, CD4, CD8, CD11b, CD11c, CD19, FcεR1, Ly6G/Ly6C, Ter119.

b) Representative flow cytometry plots showing ivCD45 labelling of the LiveCD45^{+}lineage^{+} population. Numbers within gates indicate the percentage of the parent population each gate represents.

Supplementary Figure 5

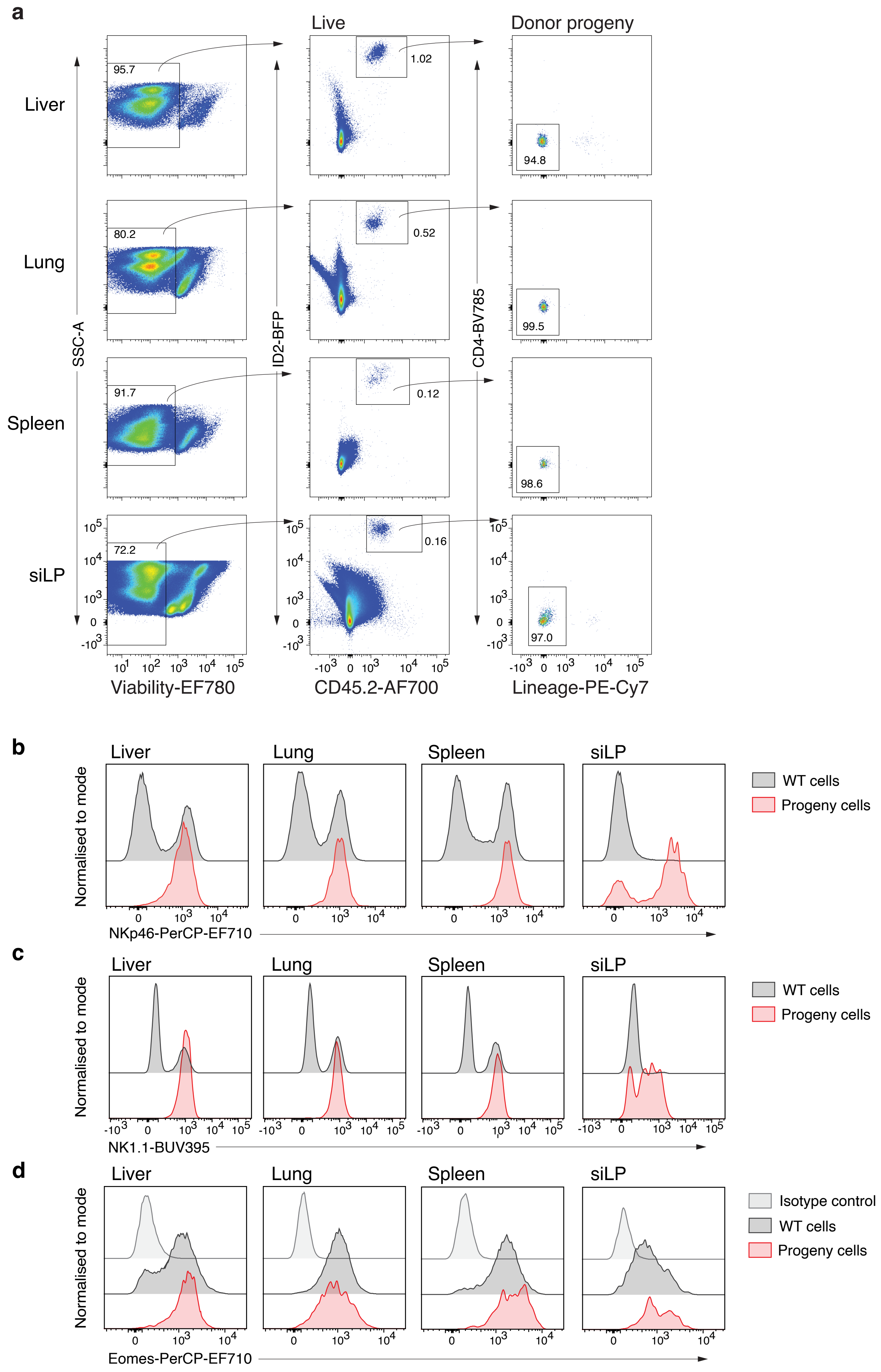

Supplementary Figure 5

Gating strategy used to define lineage negative siLP-ILCP donor progeny in vivo and definition of positive and negative populations for NKp46, NK1.1 and Eomes in the indicated tissues.

a) Gating strategy used to define lineage negative siLP-ILCP donor progeny analysed in **Fig. 3 and 4**, and **Supplementary Fig.6**. Lineage = CD3, CD8, CD19, Ter119. Numbers indicate the percentage that the gated cells constitute of the parent population.

b) Representative NKp46-PerCP-EF710 staining of LiveCD45$^+$Lineage$^-$Rorγt-Kat$^-$IL7Rα^- cells from either a wildtype animal (WT) or the siLP-ILCP donor progeny in the indicated tissues demonstrating that the positive progeny staining aligns with the positive population from the WT.

c) Representative NK1.1-BUV395 staining of LiveCD45$^+$Lineage$^-$Rorγt-Kat$^-$IL7Rα^- cells from either a wildtype animal (WT) or the siLP-ILCP donor progeny in the indicated tissues demonstrating that the positive progeny staining aligns with the positive population from the WT.

d) Representative staining for Eomes-PerCP-EF710 of LiveCD45$^+$Lineage$^-$Rorγt-Kat$^-$IL7Rα^- NK1.1$^+$ cells from either a wildtype animal (WT) or the siLP-ILCP donor progeny in the indicated tissues. Staining of the same population from WT with an isotype control which was used to set the Eomes positive/negative gate in the data analysis is also shown.

Supplementary Figure 6

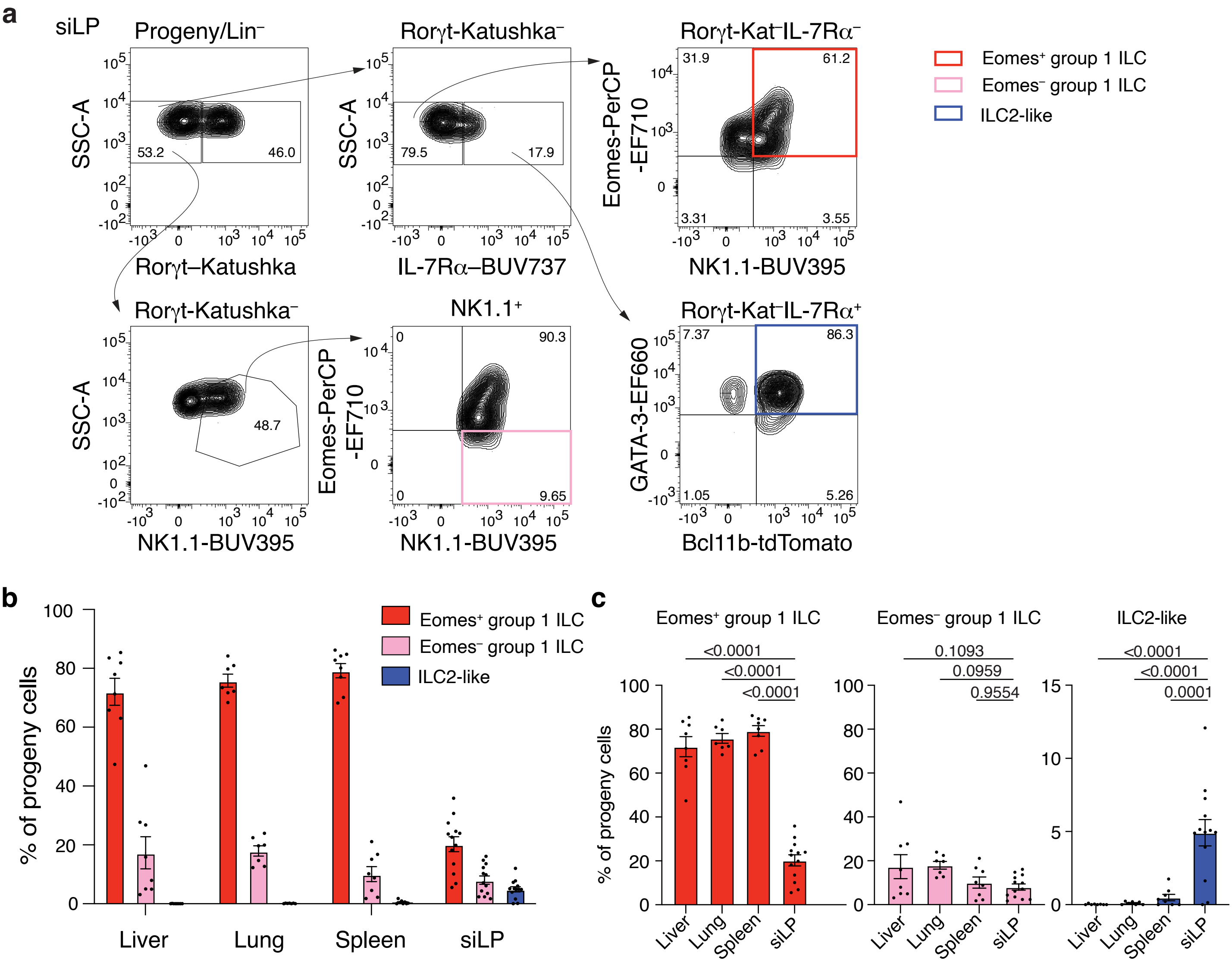

Supplementary Figure 6

Analysis of transcription factor expression indicates that donor siLP-ILCPs generate group 1 ILCs and ILC2 in the siLP of *Rag2*$^{-/-}$*Il2rg*$^{-/-}$ recipient mice

a) Representative flow cytometry plots defining the phenotype of donor progeny in the siLP of recipients with respect to expression of the transcription factors Eomes and GATA-3. Numbers within quadrants are the percentage of the parent gate. Gating strategy to define lineage negative progeny is shown in **Supplementary Fig. 5a**.

b) Quantification of the proportions of progeny in each tissue of each phenotype as defined by transcription factor expression. Eomes^{+} group 1 ILC defined as CD45.2^{+}Id2-BFP^{+}lineage^{-} Rorγt-Katushka^{-}IL-7Rα^{-}NK1.1^{+}Eomes^{+} (red), Eomes^{-} group 1 ILC defined as CD45.2^{+}Id2-BFP^{+}lineage^{-}Rorγt-Katushka^{-}NK1.1^{+}Eomes^{-} (pink) and ILC2-like defined as CD45.2^{+}Id2-BFP^{+}lineage^{-}Rorγt-Katushka^{-}IL-7Rα^{+}Bcl11b^{+}GATA-3^{hi} (blue). Data plotted as mean with SEM error bars.

c) Analysis of the statistical significance between the proportions of the progeny populations in the siLP compared with those in the liver, lung and spleen (one-way ANOVA with Tukey's multiple comparisons test). No significant differences were observed in the proportion of Eomes^{-} group 1 ILC present in the different tissues. Data are cumulative from 5 independent experiments involving a total of 8 (lung, liver, spleen) or 13 (siLP) mice and plotted as mean with SEM error bars. Source data are provided as a Source Data file.

Supplementary Figure 7

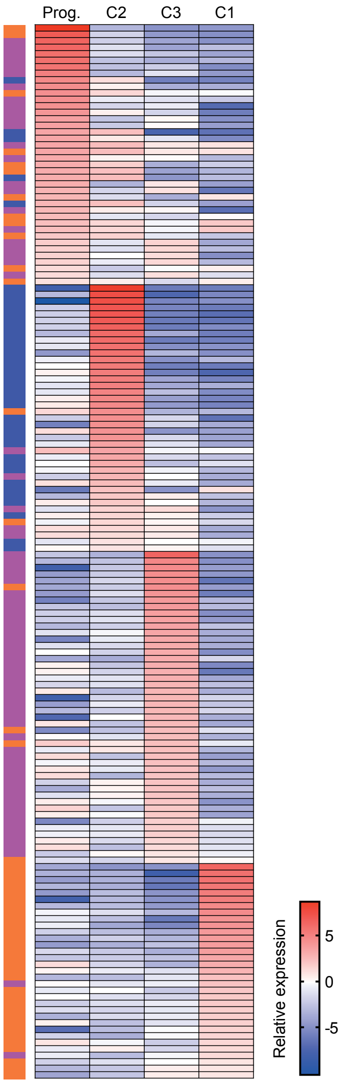

Supplementary Figure 7

Heatmap showing expression of published gene signatures for group 1, 2 and 3 ILCs across the siLP-ILCP progenitor and siLP progeny clusters (C1-C3)

Each column represents a cluster and each row a gene from published gene signatures [1,2]. Full gene list in **Supplementary Data 1**. Side bar colour codes each row according to published ILC group that the gene is attributed to; orange: group 1 ILC, blue: ILC2 and magenta: ILC3. Prog.= progenitor cluster.

Supplementary Figure 8

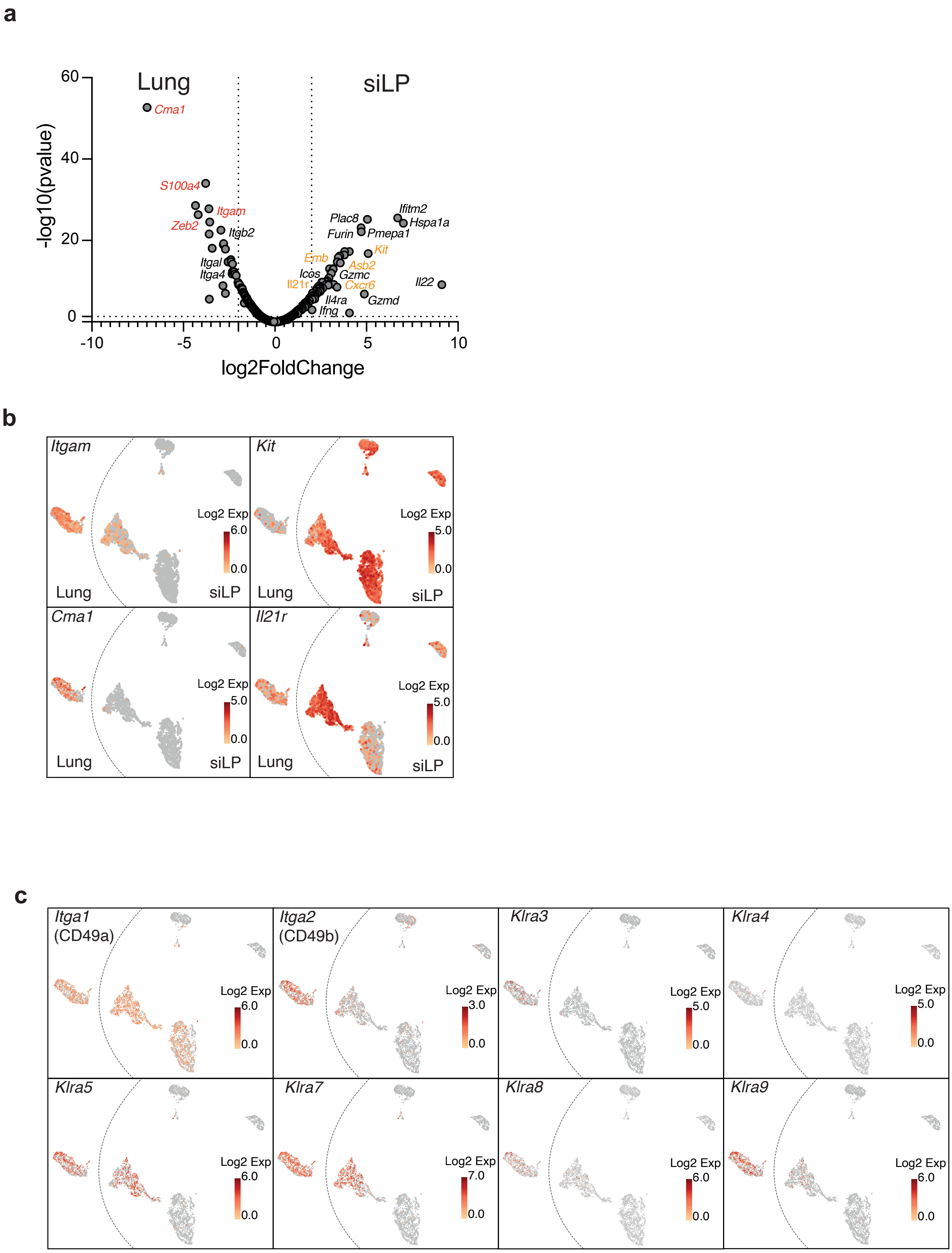

Supplementary Figure 8

scRNAseq analysis of siLP-ILCP group 1 ILC progeny identifies discrete clusters in the lung and siLP which do not fall into a clear ILC1/NK cell dichotomy

a) Volcano plot showing the scRNAseq expression analysis comparing group 1 ILC progeny of siLP-ILCPs purified from recipient lung and siLP. "Non-tissue specific" associated genes highlighted in red and "tissue specific" genes and *Il21r*, an ILC1 associated gene, highlighted in orange[3,4].

b) UMAP plot with expression level (log2 expression) of indicated genes per individual cell.

c) UMAP plot with expression level (log2 expression) of indicated genes per individual cell.

Supplementary Figure 9

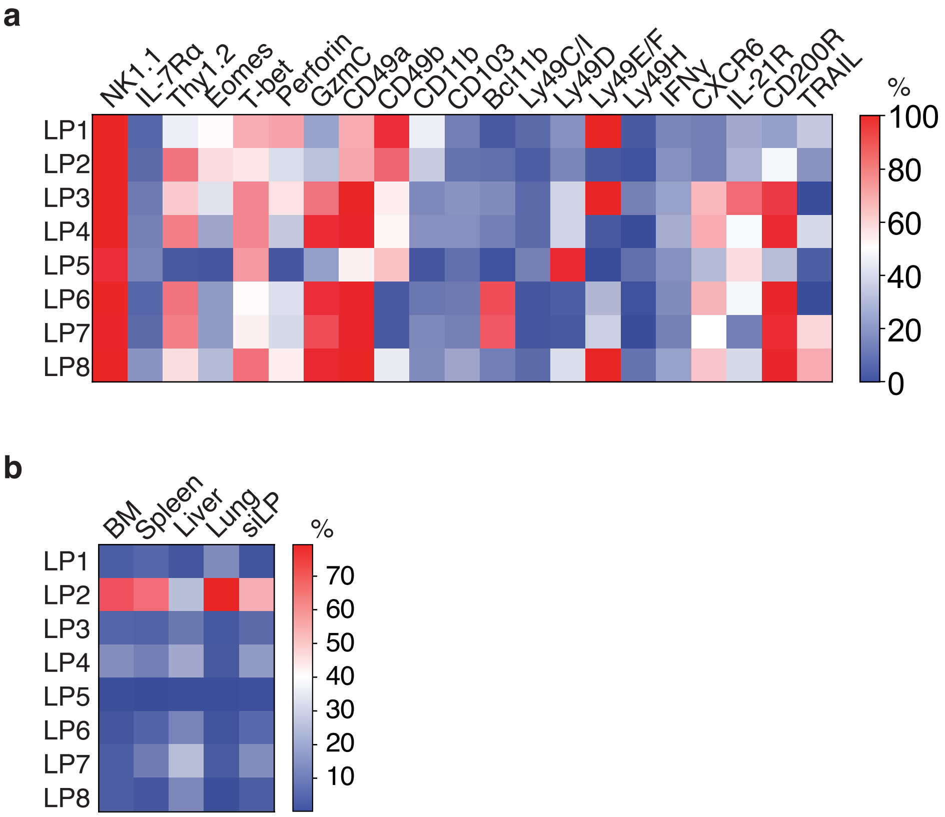

Supplementary Figure 9

Analysis of ILC1 and NK cell signature markers indicate the phenotypes of siLP-ILCP group 1 ILC progeny vary across tissues and lie on a continuum

a) Heatmap of expression of the indicated ILC1/NK cell markers within the identified clusters expressed as a percentage of cells positive for each marker.

b) Heatmap showing the relative contributions of each cluster defined by high-dimensional spectral flow cytometry (LP1-LP8) to the overall composition of the group 1 ILC progeny in the recipient tissues indicated.

Supplementary Figure 10

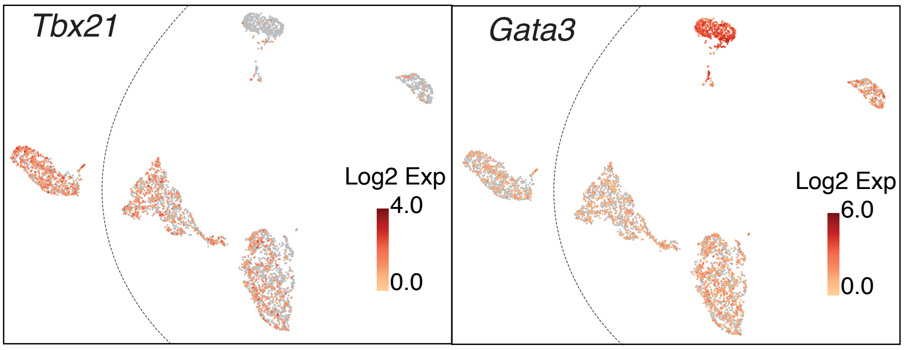

Supplementary Figure 10

Gene expression analysis of *Tbx21* and *Gata3* confirms siLP-ILCPs do not include mature group 1 ILCs or ILC2s

UMAP plot with expression level (log2 expression) of indicated genes per individual cell.

Supplementary Figure 11

a

$CD45^{+}Lin^{-}IL7R\alpha^{+}ID2\text{-}BFP^{+}NKp46^{-}KLRG1^{-}NK1.1^{-}Ror\gamma t\text{-}Kat^{-}CCR6^{-}$

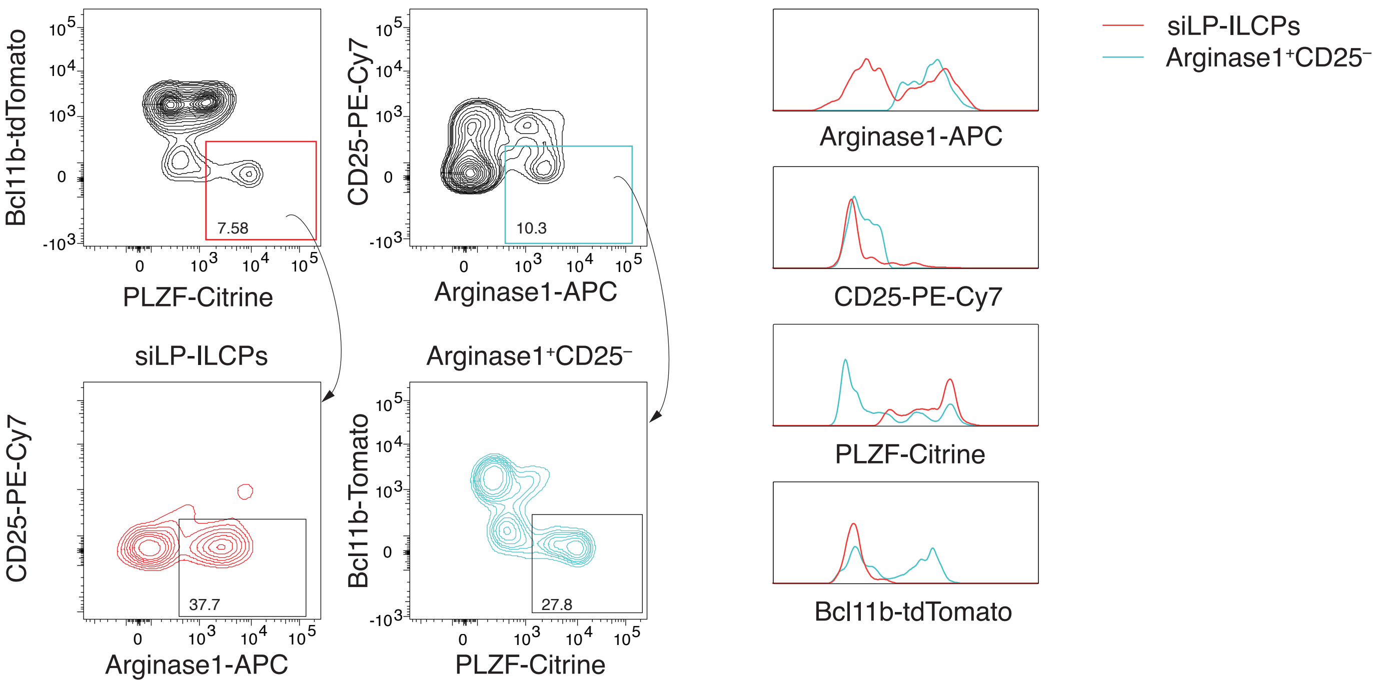

b

$CD45^{+}Lin^{-}IL7R\alpha^{+}ID2\text{-}BFP^{+}NKp46^{-}KLRG1^{-}NK1.1^{-}Ror\gamma t\text{-}Kat^{-}CCR6^{-}$

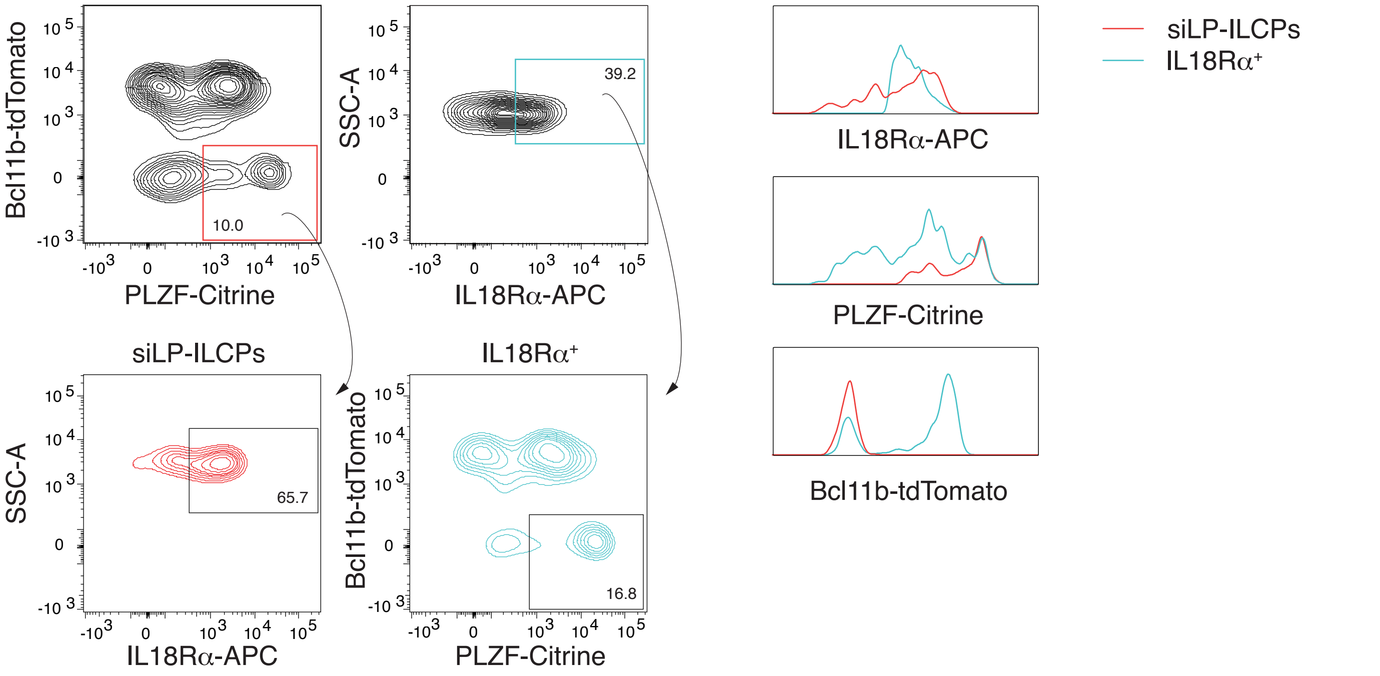

Supplementary Figure 11

Flow cytometric analysis of the expression of Arginase 1 and IL-18Rα by siLP-ILCPs.

a) Representative flow cytometry plots showing siLP-ILCP expression of Arginase 1 and CD25 and PLZF and Bcl11b expression by an Arginase 1/CD25-defined population (prior gating strategy as for **Fig. 1b**). Histograms compare the expression of these 4 key markers by these two populations. Data are concatenated from a total of 10 male and female four colour reporter mice of 14-34 weeks of age, analysed over 2 experiments. **b)** Representative flow cytometry plots showing siLP-ILCP expression of IL-18Ra and PLZF and Bcl11b expression by an IL-18Rα-defined population (prior gating strategy as for **Fig. 1b**). Histograms compare the expression of these 3 key markers by these two populations. Data are concatenated from a total of 6 male four colour reporter mice of 9-14 weeks of age and representative of 2 independent experiments. Numbers within gates indicate the percentage of the parent population each gate represents.

Supplementary Figure 12

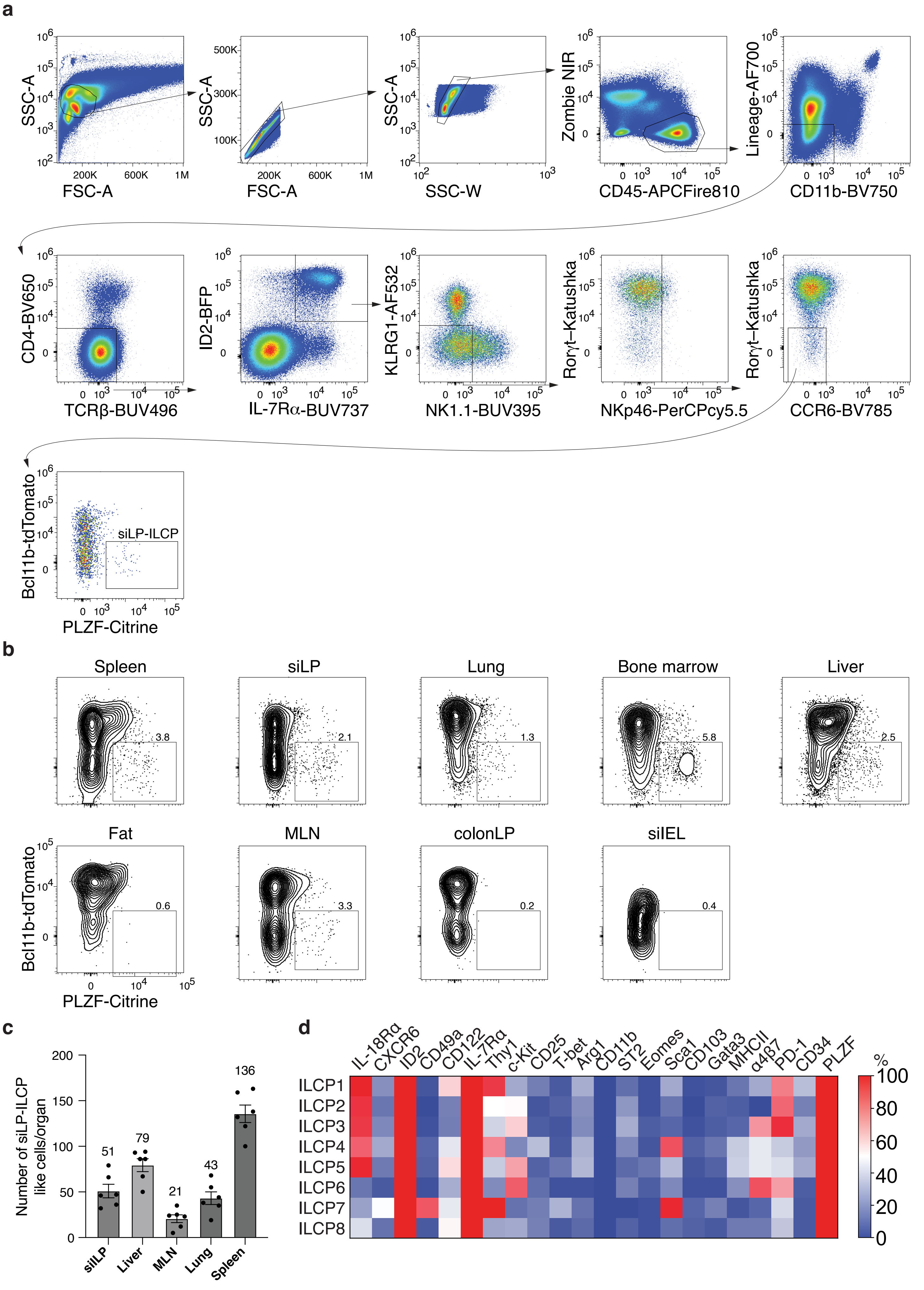

Supplementary Figure 12

High-dimensional spectral flow cytometry analysis of CD45^{+}lineage^{-}Id2-BFP^{+}IL-7Rα^{+}NK1.1^{-}NKp46^{-}KLRG1^{-}Rorγt-Katushka^{-}CCR6^{-}PLZF^{+}Bcl11b^{-} cells from siLP, BM, spleen, liver, MLN and lung.

a) Gating strategy used for high-dimensional spectral flow cytometric characterisation of siLP-ILCPs and siLP-ILCP-like cells within haematopoietic cells found across several tissues analysed in **Fig.9**. Lineage = CD3, CD8, CD11c, CD19, FcεR1, Ly6G/Ly6C and Ter119.

b) Contour plots from concatenated samples from 6 mice are presented to depict the proportion of siLP-ILCP-like cells within indicated tissues. Results are representative of one experiment (n = 6). Numbers above the PLZF-Citrine^{+}Bcl11b-tdTomato^{-} gates indicate the percentage of the parent population which is CD45^{+}lineage^{-}Id2-BFP^{+}IL-7Rα^{+}NK1.1^{-}NKp46^{-}KLRG1^{-}Rorγt-Katushka^{-}CCR6^{-} as defined in the gating strategy shown in **(a)**.

c) Quantification of the average number of CD45^{+}lineage^{-}Id2-BFP^{+}IL-7Rα^{+}NK1.1^{-}NKp46^{-}KLRG1^{-}Rorγt-Katushka^{-}CCR6^{-}PLZF^{+}Bcl11b^{-} cells (siLP-ILCP like), across the 6 mice analysed, per organ (siLP, liver, mLN, lung and spleen) per mouse. Data plotted as mean (shown above each respective bar) with SEM error bars. Source data are provided as a Source Data file.

d) Heatmap of expression of the indicated markers within the CD45^{+}lineage^{-}Id2-BFP^{+}IL-7Rα^{+}NK1.1^{-}NKp46^{-}KLRG1^{-}Rorγt-Katushka^{-}CCR6^{-}PLZF^{+}Bcl11b^{-} subclusters defined in **Fig. 9a** expressed as a percentage of cells positive for each marker.

Supplementary Table 1
Numbers of siLP-ILCPs transferred into *Rag2*$^{-/-}$*Il2rg*$^{-/-}$ recipients and progeny recovered

Experiment	Total number of donor progenitors	Number of Recipients	Total number of donor progeny recovered	Expansion expressed as population doublings
1	630	2	17329	4.782
2	1248	3	42474	5.089
3	491	3	20392	5.376

Supplementary References

1 Robinette, M. L. *et al.* Transcriptional programs define molecular characteristics of innate lymphoid cell classes and subsets. *Nat Immunol* **16**, 306-317 (2015). https://doi.org:10.1038/ni.3094

2 Gury-BenAri, M. *et al.* The Spectrum and Regulatory Landscape of Intestinal Innate Lymphoid Cells Are Shaped by the Microbiome. *Cell* **166**, 1231-1246 e1213 (2016). https://doi.org:10.1016/j.cell.2016.07.043

3 McFarland, A. P. *et al.* Multi-tissue single-cell analysis deconstructs the complex programs of mouse natural killer and type 1 innate lymphoid cells in tissues and circulation. *Immunity* **54**, 1320-1337 e1324 (2021). https://doi.org:10.1016/j.immuni.2021.03.024

4 Cortez, V. S. *et al.* Transforming Growth Factor-beta Signaling Guides the Differentiation of Innate Lymphoid Cells in Salivary Glands. *Immunity* **44**, 1127-1139 (2016). https://doi.org:10.1016/j.immuni.2016.03.007
