# Supplementary material for: Recipient tissue microenvironment determines developmental path of intestinal innate lymphoid progenitors
Source: Nat Commun. 2024 Sep 6;15:7809. doi: 10.1038/s41467-024-52155-2 (PMC11379955; doi:10.1038/s41467-024-52155-2)
Supplement: Supplementary file 3 — Description of Additional Supplementary Files [file 41467_2024_52155_MOESM3_ESM.pdf]

Description of Additional Supplementary Files

File Name: Supplementary Data 1
Description: Gene list used for Supplementary Figure 7 heatmap (in the order of the figure) with ILC subset association.

File Name: Supplementary Data 2
Description: List of flow cytometry antibodies used in the study.
