# Supplementary material for: Recipient tissue microenvironment determines developmental path of intestinal innate lymphoid progenitors
Source: Nat Commun. 2024 Sep 6;15:7809. doi: 10.1038/s41467-024-52155-2 (PMC11379955; doi:10.1038/s41467-024-52155-2)
Supplement: Supplementary file 6 — Reporting Summary [file 41467_2024_52155_MOESM6_ESM.pdf]

nature portfolio

Corresponding author(s): Andrew McKenzie and Paula Clark

Last updated by author(s): Jul 31, 2024

Reporting Summary

Nature Portfolio wishes to improve the reproducibility of the work that we publish. This form provides structure for consistency and transparency in reporting. For further information on Nature Portfolio policies, see our Editorial Policies and the Editorial Policy Checklist.

Statistics

For all statistical analyses, confirm that the following items are present in the figure legend, table legend, main text, or Methods section.

n/a	Confirmed	
☐	☒	The exact sample size (*n*) for each experimental group/condition, given as a discrete number and unit of measurement
☐	☒	A statement on whether measurements were taken from distinct samples or whether the same sample was measured repeatedly
☐	☒	The statistical test(s) used AND whether they are one- or two-sided<br>*Only common tests should be described solely by name; describe more complex techniques in the Methods section.*
☒	☐	A description of all covariates tested
☒	☐	A description of any assumptions or corrections, such as tests of normality and adjustment for multiple comparisons
☐	☒	A full description of the statistical parameters including central tendency (e.g. means) or other basic estimates (e.g. regression coefficient) AND variation (e.g. standard deviation) or associated estimates of uncertainty (e.g. confidence intervals)
☐	☒	For null hypothesis testing, the test statistic (e.g. *F*, *t*, *r*) with confidence intervals, effect sizes, degrees of freedom and *P* value noted<br>*Give P values as exact values whenever suitable.*
☒	☐	For Bayesian analysis, information on the choice of priors and Markov chain Monte Carlo settings
☒	☐	For hierarchical and complex designs, identification of the appropriate level for tests and full reporting of outcomes
☒	☐	Estimates of effect sizes (e.g. Cohen's *d*, Pearson's *r*), indicating how they were calculated

Our web collection on statistics for biologists contains articles on many of the points above.

Software and code

Policy information about availability of computer code

Data collection	BD LSRFortessa Special Order (5 laser), BD<br>iCyt Synergy, Sony Biotechnology SY3200<br>ID7000 spectral cell analyser (Sony)<br>BD FACS Aria Fusion Special Order System (BD Biosciences)<br>Illumina NovaSeq
Data analysis	Prism 10, GraphPad Prism<br>10x Cell Ranger (version 6.0.1)<br>STAR aligner (version 2.7.2a)<br>10x Genomics Loupe Browser<br>FlowJo. FlowJo, LLC, v10, RRID: SCR_008520<br>BD FACSDiva Software V6.2

For manuscripts utilizing custom algorithms or software that are central to the research but not yet described in published literature, software must be made available to editors and reviewers. We strongly encourage code deposition in a community repository (e.g. GitHub). See the Nature Portfolio guidelines for submitting code & software for further information.

Data

Policy information about availability of data

All manuscripts must include a data availability statement. This statement should provide the following information, where applicable:

- Accession codes, unique identifiers, or web links for publicly available datasets
- A description of any restrictions on data availability
- For clinical datasets or third party data, please ensure that the statement adheres to our policy

All sequencing data generated in this study have been deposited with the Gene Expression Omnibus (GEO) under accession number GSE234835. Previously published sequencing data for the aceNKP progeny are from GSE213814. All other data are available in the article and its Supplementary files or from the corresponding author upon request. Source data are provided with this paper.

Research involving human participants, their data, or biological material

Policy information about studies with human participants or human data. See also policy information about sex, gender (identity/presentation), and sexual orientation and race, ethnicity and racism.

Reporting on sex and gender	*Use the terms sex (biological attribute) and gender (shaped by social and cultural circumstances) carefully in order to avoid confusing both terms. Indicate if findings apply to only one sex or gender; describe whether sex and gender were considered in study design; whether sex and/or gender was determined based on self-reporting or assigned and methods used. Provide in the source data disaggregated sex and gender data, where this information has been collected, and if consent has been obtained for sharing of individual-level data; provide overall numbers in this Reporting Summary. Please state if this information has not been collected. Report sex- and gender-based analyses where performed, justify reasons for lack of sex- and gender-based analysis.*
Reporting on race, ethnicity, or other socially relevant groupings	*Please specify the socially constructed or socially relevant categorization variable(s) used in your manuscript and explain why they were used. Please note that such variables should not be used as proxies for other socially constructed/relevant variables (for example, race or ethnicity should not be used as a proxy for socioeconomic status). Provide clear definitions of the relevant terms used, how they were provided (by the participants/respondents, the researchers, or third parties), and the method(s) used to classify people into the different categories (e.g. self-report, census or administrative data, social media data, etc.) Please provide details about how you controlled for confounding variables in your analyses.*
Population characteristics	*Describe the covariate-relevant population characteristics of the human research participants (e.g. age, genotypic information, past and current diagnosis and treatment categories). If you filled out the behavioural & social sciences study design questions and have nothing to add here, write "See above."*
Recruitment	*Describe how participants were recruited. Outline any potential self-selection bias or other biases that may be present and how these are likely to impact results.*
Ethics oversight	*Identify the organization(s) that approved the study protocol.*

Note that full information on the approval of the study protocol must also be provided in the manuscript.

Field-specific reporting

Please select the one below that is the best fit for your research. If you are not sure, read the appropriate sections before making your selection.

☒ Life sciences ☐ Behavioural & social sciences ☐ Ecological, evolutionary & environmental sciences

For a reference copy of the document with all sections, see nature.com/documents/nr-reporting-summary-flat.pdf

Life sciences study design

All studies must disclose on these points even when the disclosure is negative.

Sample size	No statistical methods were used to determine sample size. Sample size was determined to be adequate based on the reproducibility between independent experiments. Sample size was also based on field standards to avoid unethical use of mice. Sample sizes were as described in the figure legends.
Data exclusions	No data exclusion
Replication	Experiments were repeated multiple times as stated in the figure legends
Randomization	No randomisation was performed. Where the outcomes for two separate groups of mice were analysed (BM-ILCPs vs siLP-ILCPs or siLP-ILCP growth on OP9 vs OP9-DL cells) mice used for each group were matched for gender and the mix of ages.
Blinding	No blinding was performed as all samples were processed together identically and analysed by objective measurements (FACS scRNAseq)

Blinding

Reporting for specific materials, systems and methods

We require information from authors about some types of materials, experimental systems and methods used in many studies. Here, indicate whether each material, system or method listed is relevant to your study. If you are not sure if a list item applies to your research, read the appropriate section before selecting a response.

Materials & experimental systems

n/a	Involved in the study
☐	☒ Antibodies
☐	☒ Eukaryotic cell lines
☒	☐ Palaeontology and archaeology
☐	☒ Animals and other organisms
☒	☐ Clinical data
☒	☐ Dual use research of concern
☒	☐ Plants

Methods

n/a	Involved in the study
☒	☐ ChIP-seq
☐	☒ Flow cytometry
☒	☐ MRI-based neuroimaging

Antibodies

Antibodies used

Anti-mouse CD11c N418 Alexa Fluor 700 eBioscience 56-0114-82 1/200 AB_493992
Anti-mouse CD11c N418 PE-Cy7 eBioscience 25-0114-82 1/500 AB_469590
Anti-mouse CD19 eBio1D3 PE-Cy7 eBioscience 25-0193-82 1/500 AB_657663
Anti-mouse CD25 PC61.5 PE-Cy7 eBioscience 25-0251-82 1/500 AB_469608
Anti-mouse CD3 17A2 Alexa Fluor 700 eBioscience 56-0032-82 1/200 AB_529507
Anti-mouse CD34 RAM34 eFluor 660 eBioscience 50-0341-82 1/300 AB_10596826
Anti-mouse CD4 GK1.5 Alexa Fluor 700 eBioscience 56-0041-82 1/200 AB_493999
Anti-mouse CD45 30-F11 APC eBioscience 17-0451-83 1/500 AB_469393
Anti-mouse CD45.2 104 Alexa Fluor 700 eBioscience 56-0454-82 1/200 AB_657752
Anti-mouse CD8a 53-6.7 PE-Cy7 eBioscience 25-0081-82 1/500 AB_469584
Anti-mouse Eomes Dan11mag PE eBioscience 12-4875-82 1/500 AB_1603275
Anti-mouse Eomes Dan11mag PE-Cy7 eBioscience 25-4875-80 1/300 AB_2573453
Anti-mouse Eomes Dan11mag PerCP-EF710 eBioscience 46-4875-82 1/1000 AB_10597455
Anti-mouse FceR1 MAR-1 PE-Cy7 eBioscience 25-5898-82 1/500 AB_2573493
Anti-mouse Integrin a4b7 (LPAM-1) DATK-32 PerCP-EF710 eBioscience 46-5887-82 1/200 AB_2573793
Anti-mouse KLRG1 2F1 AF532 eBioscience 58-5893-80 1/200 AB_2815281
Anti-mouse KLRG1 2F1 APC eBioscience 17-5893-82 1/300 AB_469469
Anti-mouse KLRG1 2F1 PerCP-EF710 eBioscience 46-5893-82 1/500 AB_10670282
Anti-mouse Ly-49E/F CM4 APC eBioscience 17-5848-80 1/200 AB_10557243
Anti-mouse Ly-6G/Ly-6C RB6-8C5 Alexa Fluor 700 eBioscience 56-5931-82 1/200 AB_494007
Anti-mouse Ly-6G/Ly-6C RB6-8C5 PE-Cy7 eBioscience 25-5931-82 1/500 AB_469663
Anti-mouse Ly6A/E (Sca-1) D7 PE-Cy7 eBioscience 25-5981-82 1/500 AB_469669
Anti-mouse NK1.1 PK136 Alexa Fluor 700 eBioscience 56-5941-82 1/200 AB_2574505
Anti-mouse NKp46 29A1.4 eFluor 660 eBioscience 50-3351-82 1/200 AB_10598664
Anti-mouse NKp46 29A1.4 PerCP-EF710 eBioscience 46-3351-82 1/200 AB_1834441
Anti-mouse TER-119 TER-119 PE-Cy7 eBioscience 25-5921-82 1/500 AB_469661
Anti-mouse/human Arginase 1 A1exF5 APC eBioscience 17-3697-82 1/200 AB_2734835
Anti-mouse/human Arginase 1 A1exF5 BV711 eBioscience 407-3697-80 1/200 AB_3074093
Anti-mouse/human Gata-3 TWAJ PE-eFluor610 eBioscience 61-9966-41 1/200 AB_2574685
Anti-mouse/human/porcine Gata-3 TWAJ eFluor 660 eBioscience 50-9966-42 1/200 AB_10596663
Anti-mouse/rat/ rhesus monkey CD278 (ICOS) C398.4A APC eBioscience 17-9949-82 1/500 AB_11149880
Anti-mouse CD200R OX110 PerCP-EF710 eBioscience 46-5201-82 1/300 AB_10804765
Anti-mouse CCR6 29-2L17 BV785 BioLegend 129823 1/200 AB_2715923
Anti-mouse CD103 QA17A24 PE-Fire810 Biolegend 156919 1/300 AB_2924488
Anti-mouse CD11b M1/70 PE-Cy7 BioLegend 101216 1/500 AB_312799
Anti-mouse CD127/IL7Ra SB/199 Biotin BioLegend 121104 1/300 AB_493502
Anti-mouse CD186 (CXCR6) SA051D1 APC-Fire 750 Biolegend 151130 1/200 AB_2910304
Anti-mouse CD186 (CXCR6) SA051D1 BV711 Biolegend 151111 1/100 AB_2721558
Anti-mouse CD19 6D5 Alexa Fluor 700 BioLegend 115528 1/200 AB_493735
Anti-mouse CD196 (CCR6) 29-2L17 APC BioLegend 129814 1/200 AB_1877147
Anti-mouse CD25 PC61 BV510 BioLegend 102042 1/200 AB_2562269
Anti-mouse CD253 (TRAIL) N2B2 PerCP-Cy5.5 Biolegend 109314 1/100 AB_2721720
Anti-mouse CD279 (PD-1) 29F.1A12 PerCP-Fire806 BioLegend 135261 1/200 AB_2941420
Anti-mouse CD335 (NKp46) 29A1.4 PerCP-Cy5.5 BioLegend 137610 1/100 AB_10641137
Anti-mouse CD335 (NKp46) 29A1.4 PerCP-Fire806 Biolegend 137649 1/200 AB_2941422
Anti-mouse CD3e 145-2C11 BV605 Biolegend 100351 1/200 AB_2565842
Anti-mouse CD3e 145-2C11 PE-Cy7 BioLegend 100320 1/500 AB_312684
Anti-mouse CD4 GK1.5 BV605 Biolegend 100451 1/200 AB_2564591

Anti-mouse CD4 GK1.5 BV650 BioLegend 100469 1/300 AB_2783035
Anti-mouse CD4 RM4-5 BV785 BioLegend 100551 1/300 AB_11218992
Anti-mouse CD4 GK1.5 PE-Cy7 BioLegend 100422 1/500 AB_312706
Anti-mouse CD45 30-F11 APC-Fire810 BioLegend 103174 1/300 AB_2860600
Anti-mouse CD45 30-F11 BV510 BioLegend 103138 1/200 AB_2563061
Anti-mouse CD45.2 104 BV421 Biolegend 109832 1/200 AB_2565511
Anti-mouse CD49b (Integrin alpha 2) DX5 PE-Dazzle 594 Biolegend 108924 1/200 AB_2565271
Anti-mouse CD62L MEL-14 BV570 Biolegend 104433 1/500 AB_10900262
Anti-mouse CD8a 53-6.7 Alexa Fluor 700 BioLegend 100730 1/200 AB_493703
Anti-mouse CD8a 53-6.7 BV605 Biolegend 100744 1/200 AB_2562609
Anti-mouse FceR1 MAR-1 Alexa Fluor 700 BioLegend 134324 1/200 AB_2566734
Anti-mouse Granzyme C SFC1D8 PE Biolegend 150803 1/2000 AB_2566429
Anti-mouse IFNg XMG1.2 BV785 BioLegend 505838 1/200 AB_11219004
Anti-mouse IL-21R 4A9 PE-Cyanine5 Biolegend 131908 1/200 AB_2124134
Anti-mouse IL18Ra A17071D APC BioLegend 157906 1/200 AB_2860735
Anti-mouse MHCII (I-A/I-E) M5/114.15.2 PerCP BioLegend 107623 1/300 AB_893586
Anti-mouse Perforin S16009B APC BioLegend 154404 1/1000 AB_2721465
Anti-mouse Perforin S16009A APC-Fire750 Biolegend 154317 1/200 AB_2924481
Anti-mouse TCRb H57-597 BV605 Biolegend 109241 1/200 AB_2629563
Anti-mouse TCRgd GL3 BV605 Biolegend 118129 1/200 AB_2563356
Anti-mouse TER-119 TER-119 Alexa Fluor 700 BioLegend 116220 1/200 AB_528963
Anti-mouse/cynomolgus/human/rhesus CD11b M1/70 BV750 Biolegend 101267 1/200 AB_2810328
Anti-mouse/human KLRG1 2F1/KLRG1 BV510 Biolegend 138421 1/200 AB_2563800
Anti-mouse/human T-bet 4B10 BV605 BioLegend 644817 1/300 AB_11219388
Anti-mouse CD117 (c-Kit) 2B8 BV480 BD Biosciences 566081 1/300 AB_2739493
Anti-mouse CD45 30-F11 BUV395 BD Biosciences 564279 1/200 AB_2651134
Anti-mouse CD90.2 (Thy1.2) 30-H12 BUV805 BD Biosciences 741909 1/300 AB_2871223
Anti-Mouse Ly-49C and Ly49I 5E6 BUV615 BD Biosciences 751641 1/100 AB_2875634
Anti-Mouse Ly-49D 4E5 BUV496 BD Biosciences 749990 1/300 AB_2874212
Anti-Mouse Ly-49H 3D10 BUV661 BD Biosciences 750512 1/300 AB_2874666
Anti-mouse NK1.1 PK136 BUV395 BD Biosciences 564144 1/200 AB_2738618
Anti-mouse TCRb H57-597 BUV496 BD Biosciences 749915 1/200 AB_2874154
Anti-mouse/human T-bet O4-46 RB780 BD Biosciences 569089 1/100
Anti-mouse/rat CD49a Ha31/8 BUV563 BD Biosciences 741306 1/300 AB_2870831
Anti-ICOS (CD278) C398.4A BV480 BD Bioscience 566085 1/300
Anti-Mouse CD122 TM-β1 BUV615 BD Bioscience 751254 1/200 AB_2875271
Anti-mouse T1/ST2 DJ8 FITC MD Bioproducts 101001F 1/100 AB_947549
Streptavidin BUV737 BD Biosciences 564293 1/300 AB_2869560

Validation

All antibodies used in this study were commercially available and validation statements can be found on the manufacturer's website.

Eukaryotic cell lines

Policy information about cell lines and Sex and Gender in Research

Cell line source(s)

OP9 and OP9-DL1, Sunnybrook Research Institute, Schmitt and Zúñiga-Pflücker, 2002
B16F10 melanoma cell line CRL6475 ATCC

Authentication

Authentication was not performed.

Mycoplasma contamination

Tested negative.

Commonly misidentified lines
(See ICLAC register)

Not used in the study.

Animals and other research organisms

Policy information about studies involving animals; ARRIVE guidelines recommended for reporting animal research, and Sex and Gender in Research

Laboratory animals

C57BL/6Jola, Jackson Labs (Bred in LMB), RRID: IMSR_JAX:000664
CD45.1 Rag2−/−Il2rgc−/−, Institute Pasteur, Serafini et al., 2014
PLZF-Citrine generated at the MRC-LMB Rodriguez-Rodriguez et al 2022
Bcl11b-tdTomato gift of Pentau Liu Li et al 2010
Rorgt-Katushka generated at the MRC-LMB Walker et al 2019
ID2-BFP generated at the MRC-LMB Walker et al 2019
Four colour reporter mice were generated by interbreeding the individual reporter strains.
Four colour reporter mice used were 7-30 weeks of age
CD45.1 Rag2−/−Il2rgc−/− used were of 6-23 weeks of age

Wild animals

Study did not involve wild animals.

Reporting on sex

siLP-ILCPs from both sexes were analysed across multiple experiments. For ILCP transfer experiments ILCPs from a single sex were pooled and where the donors were female these were transferred into male and female recipients. Where donors were male only male recipients were used.

Field-collected samples

The study did not involve samples collected from the field.

Ethics oversight

All experiments undertaken in this study were done so with the approval of the LMB Animal Welfare and Ethical Review Body (AWERB) and of the UK Home Office.

Note that full information on the approval of the study protocol must also be provided in the manuscript.

Plants

Seed stocks

Report on the source of all seed stocks or other plant material used. If applicable, state the seed stock centre and catalogue number. If plant specimens were collected from the field, describe the collection location, date and sampling procedures.

Novel plant genotypes

Describe the methods by which all novel plant genotypes were produced. This includes those generated by transgenic approaches, gene editing, chemical/radiation-based mutagenesis and hybridization. For transgenic lines, describe the transformation method, the number of independent lines analyzed and the generation upon which experiments were performed. For gene-edited lines, describe the editor used, the endogenous sequence targeted for editing, the targeting guide RNA sequence (if applicable) and how the editor was applied.

Authentication

Describe any authentication procedures for each seed stock used or novel genotype generated. Describe any experiments used to assess the effect of a mutation and, where applicable, how potential secondary effects (e.g. second site T-DNA insertions, mosiacism, off-target gene editing) were examined.

Flow Cytometry

Plots

Confirm that:

☒ The axis labels state the marker and fluorochrome used (e.g. CD4-FITC).

☒ The axis scales are clearly visible. Include numbers along axes only for bottom left plot of group (a 'group' is an analysis of identical markers).

☒ All plots are contour plots with outliers or pseudocolor plots.

☒ A numerical value for number of cells or percentage (with statistics) is provided.

Methodology

Sample preparation

Cell suspensions of spleen, mesenteric lymph node (MLN) and liver were obtained by passing the tissues through a 70 m strainer. Lung tissue was pre-digested with 750 U/ml collagenase I (Gibco) and 0.3 mg/ml DNaseI (Sigma-Aldrich) prior to obtaining a single cell suspension. Bone marrow (BM) cells were removed from femurs and tibiae by centrifuging briefly at 6000 x g. For lung, liver, BM and spleen cell suspensions, red blood cells were removed by incubation with RBC lysis solution (140 mM NH4Cl, 17 mM Tris; pH 7.2). Lung lymphocytes were further enriched by centrifugation in 30% Percoll at 800 x g (GE Healthcare) whilst liver lymphocytes were enriched in 40% Percoll at 690 x g.

Blood leukocytes were prepared from 100-500 l of blood collected in EDTA-treated collection vials. Red blood cells were lysed by incubation with 25ml RBC lysis solution (140 mM NH4Cl, 17 mM Tris; pH 7.2) for 10 minutes at room temperature and leukocytes washed and filtered through a 70 m strainer.

For preparation of siLP and colonic LP lymphocytes, intestinal contents were removed by the application of gentle pressure along the length of the intestine. Intestines were opened longitudinally, cut into 3 cm long pieces and washed briefly by vortexing in PBS + 10 mM HEPES (PBS/HEPES). Epithelial cells were removed by incubation with RPMI supplemented with 2% FCS, 1 mM dithiothreitol and 5 mM EDTA for 2 x 20 mins at 37oC with shaking (200 rpm). Where appropriate small intestinal intra epithelial lymphocytes (siIELs) were collected with the epithelial fraction at this point. Intestinal pieces were washed with PBS/HEPES and incubated, with shaking, at 37oC with RPMI + 2% FCS, 0.125 KU/ml DNaseI (Sigma-Aldrich) and 62.5 g/ml Liberase TL (Roche) until no large pieces of intestine remained. Cells were then passed through a 70 m strainer, pelleted and separated over a 40%:80% gradient of Percoll at 600 x g for 20 minutes. LP lymphocytes were isolated from the interface and prepared for flow cytometric analysis. Unless stated otherwise, small intestine and colonic lamina propria (siLP and cLP) includes associated Peyer’s patches.

Cell suspensions from adipose tissue were obtained by mechanical dissociation in RPMI-1640, and digested with collagenase I (Life Technologies), DNase I (Roche) at 37oC whilst shaking. Initial wash steps were performed with PBS 3% FCS warmed to 37oC and centrifugation steps (400 x g) were performed at room temperature to allow separation of the cell pellet from the fat.

Instrument

LSRFortessa system (BD Biosciences) for analysis
iCyt Synergy system (70-um nozzle, Sony Biotechnology) for cell sorting.
ID7000 spectral cell analyser (Sony) for analysis
BD FACS Aria Fusion Special Order System (BD Biosciences) for cell sorting

Software	FACSDiva sofware (version 6.2, BD Biosciences)<br>FlowJo. FlowJo, LLC, v10, RRID: SCR_008520<br>ID7000 Software Version 2.0.2.17121 Sony
Cell population abundance	We sorted approximately 30-120 siLP-ILCPs per entire small intestine of each mouse
Gating strategy	Cells are defined in FSC/SCC plot and dead cells excluded by viability dye. Positive populations are defined by comparison to unstained controls, isotype controls or fluorescence minus one controls.<br>Multiple gating strategies employed, all are illustrated in figures or supplementary figures.

☒ Tick this box to confirm that a figure exemplifying the gating strategy is provided in the Supplementary Information.
